# A NET-competent neutrophil subpopulation drives early brain injury after subarachnoid hemorrhage

**DOI:** 10.3389/fnins.2026.1923057

**Published:** 2026-08-27

**Authors:** Kaikai Wang, Changming Liu, Haifeng Chu, Yi Huang, Guangmin Qiu, Yuchun Liu

**Affiliations:** 1 Department of Neurosurgery, The Second Affiliated Hospital, School of Medicine, Zhejiang University, Hangzhou, China; 2 Zhejiang Key Laboratory of Research and Transformation for Major Neurological Diseases, Hangzhou, China; 3 Department of Neurosurgery, The Traditional Chinese Medicine Hospital of Linping District, Hangzhou, China; 4 Ningbo Key Laboratory of Nervous System and Brain Function, Department of Neurosurgery, The First Affiliated Hospital of Ningbo University, Ningbo, China

**Keywords:** early brain injury, neuroinflammation, neutrophil extracellular traps, single-cell RNA sequencing, subarachnoid hemorrhage

## Abstract

**Introduction:**

Early brain injury (EBI) following subarachnoid hemorrhage (SAH) critically determines neurological outcomes, yet the cellular drivers remain unclear.

**Methods:**

To systematically dissect acute pathogenic programs, we performed single-cell RNAsequencing of CD45^+^ immune cells isolated from a mouse model of SAH, coupled with weighted gene co-expression network analysis (WGCNA).

**Results:**

This revealed a 24-h-specific injury module enriched for inflammatory and myeloid-related pathways. Mapping this module onto the single-cell landscape identified neutrophils as the principal cellular mediators. High-resolution subclustering further defined a distinct neutrophil subset, N_04, as the primary carrier of the 24-h pathogenic signature. N_04 is transcriptionally primed for Neutrophil Extracellular Trap (NET) formation, with key effectors including Padi4, Mmp8, and Lcn2, and spatial mapping localized N_04 to regions adjacent to the puncture site. Transcriptional regulatory analysis identified Padi4, Nfe2, and Cebpe as central nodes sustaining N_04 effector identity, while *in vivo* Padi4 deficiency or NET degradation markedly alleviated neurological deficits. Pseudotime and computational perturbation analyses indicated that N_04 represents a terminal effector state orchestrating hyper-inflammatory, degranulation, and stress-response programs. Intercellular communication modeling further positioned N_04 as a signaling hub, mediating bidirectional crosstalk with macrophages via Cxcl, Ccl, and APP pathways.

**Discussion:**

Collectively, these findings define a NET-competent neutrophil subpopulation as a central driver of acute post-SAH brain injury and nominate Padi4-dependent NETosis as a potential therapeutic target.

## Introduction

Subarachnoid hemorrhage (SAH) remains a devastating neurological emergency with high early mortality and long-term disability (Claassen and Park, 2022). Although the initial bleed causes immediate mechanical and vascular injury, much of the subsequent neurological deterioration is thought to arise from early brain injury (EBI), a complex cascade that unfolds within the first 72 h after ictus (Rass and Helbok, 2019). Neuroinflammation is widely considered one of the central mechanisms driving secondary injury during this phase (Ahn et al., 2019; Cahill et al., 2006; Zheng and Wong, 2017). Increasing evidence suggests that this period is accompanied by rapid remodeling of the neuroimmune landscape (Schneider et al., 2018), involving both resident CNS immune populations and infiltrating peripheral leukocytes (Li and Barres, 2018). However, how these early immune responses are organized in time and space—and which cell states are most directly linked to tissue-destructive programs—remains incompletely understood (Liesz et al., 2015).

This uncertainty may help explain why broad anti-inflammatory strategies have yielded limited clinical benefit after SAH (de Oliveira Manoel and Macdonald, 2018). Rather than reflecting a uniform inflammatory process, EBI is likely shaped by heterogeneous immune responses that vary across cell types, activation states, and temporal windows (Iadecola and Anrather, 2011; Ohashi et al., 2023). Conventional transcriptomic and histological approaches have been instrumental in documenting post-SAH inflammation (Mrdjen et al., 2018), but they have lacked the resolution to distinguish broadly activated immune compartments from discrete pathogenic cell states with disproportionate injurious potential (Wu F. et al., 2021). A key unresolved question, therefore, is which immune populations and cellular states dominate the acute pathogenic response during the hyper-acute phase after SAH (Zhang et al., 2023).

Among infiltrating leukocytes, neutrophils are especially compelling candidates to shape the hyper-acute phase of injury (Jickling et al., 2015). They accumulate rapidly after CNS insult, release proteases and oxidants, and can promote vascular dysfunction, blood-brain barrier disruption, and secondary neuronal damage (Friedrich et al., 2011). Yet in SAH, neutrophils have often been viewed as a relatively homogeneous population of short-lived first responders (Ng et al., 2019). This perspective may obscure the existence of specialized neutrophil states with distinct effector programs and outsized pathological influence (Xie et al., 2020). Defining whether such states emerge after SAH, when they peak, and how they engage the surrounding immune microenvironment is essential for understanding how the initial inflammatory surge is translated into acute brain injury (Li et al., 2020).

Here, we combined longitudinal single-cell RNA sequencing of CD45^+^ immune cells with spatial transcriptomics in a mouse model of SAH to resolve the temporal and anatomical logic of the acute immune response. Specifically, we sought to map the heterogeneous immune cell states during the hyper-acute phase, identify specific pathogenic subclusters associated with early brain injury, and evaluate whether targeting key neutrophil effector programs could alleviate acute neurological deficits. By systematically integrating single-cell resolution with spatial organization and functional validation, this study aims to refine the cellular framework of post-SAH neuroinflammation and inform cell-state-targeted therapeutic strategies during the critical early window after hemorrhage.

## Results

### A 24-h post-SAH injury module defines the dominant early pathogenic program

To systematically decode the temporal transcriptomic landscape following subarachnoid hemorrhage (SAH), we performed single-cell RNA sequencing (scRNA-seq) on CD45^+^ immune cells isolated from the ipsilateral hemispheres of mice at key intervals: Sham (*n* = 1), 24 h (*n* = 2), and 72 h (*n* = 2) post-injury. After rigorous quality control, a total of 32,368 high-quality cells were retained for downstream analysis.

**FIGURE 1:**
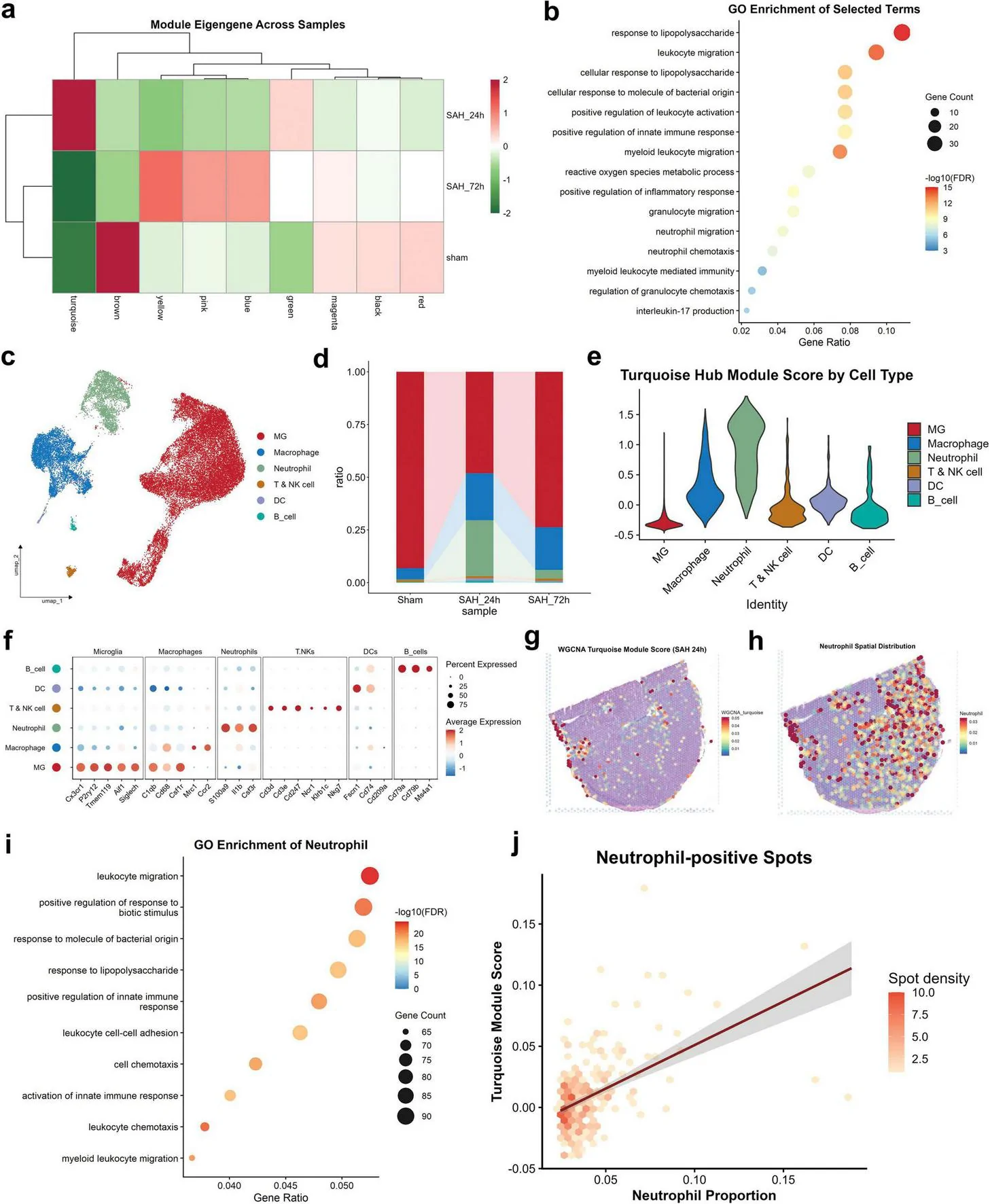
A 24-h WGCNA injury module reveals neutrophils as the principal cellular carriers of early post-SAH inflammation. **(a)** WGCNA module eigengene expression across Sham, 24 h and 72 h post-SAH samples. **(b)** GO enrichment analysis of the 24 h-associated Turquoise module. **(c,d)** UMAP visualization and proportional distribution of major CD45^+^ immune-cell populations across time points. **(e,f)** Projection of Turquoise module scores and canonical marker expression (normalized expression values) onto the single-cell immune atlas. **(g,h)** Spatial distribution of Turquoise module activity and RCTD-inferred neutrophil abundance (represented by RCTD deconvolution weights) in 24 h post-SAH tissue. **(i)** GO enrichment of neutrophil marker genes. **(j)** Correlation between spatial neutrophil abundance and Turquoise module scores across selected spots. Dot size denotes gene count and color indicates enrichment significance where applicable.

We first employed Weighted Gene Co-expression Network Analysis (WGCNA) (Langfelder and Horvath, 2008) to identify time-specific gene modules characterizing SAH pathology evolution (Figure 1a and Supplementary Figures 1a,b), further validating their disease association via module-trait correlation (Supplementary Figure 1c). Our analysis revealed three distinct modules highly correlated with specific time points: a “Brown” module associated with the Sham state, a “Turquoise” module uniquely enriched at the 24 h acute phase, and a “Yellow” module peaking at 72 h (Figure 1a). Gene Ontology (GO) enrichment analysis of these modules provided a functional map of the post-SAH transition. The Brown module (Sham-specific) was predominantly enriched in terms related to homeostatic CNS maintenance, including gliogenesis, glial cell differentiation, and the regulation of neurogenesis (Supplementary Figure 1f). In contrast, the Yellow module (72 h-specific) showed enrichment in sensory system development and the regulation of muscle contraction, particularly smooth muscle contraction (Supplementary Figure 1d), likely reflecting late-stage vascular remodeling or compensatory responses in the subacute phase (Wang et al., 2019).

Crucially, the Turquoise module, which peaked at the 24 h mark, was uniquely characterized by acute inflammatory responses, leukocyte chemotaxis, and myeloid-mediated immunity (Figure 1b), suggesting that the 24 h post-SAH window represents a critical peak for pathogenic program activation. To identify the cellular major cellular carriers of this 24 h injury module, we performed unsupervised clustering on the integrated scRNA-seq dataset, which partitioned the immune landscape into six major lineages: microglia (MG), macrophages, neutrophils, T & natural killer (NK) cells, dendritic cells (DCs), and B cells (Figures 1c,f and Supplementary Figure 1g; Garcia-Bonilla et al., 2024). Analysis of cellular dynamics across the three time points revealed a striking and transient expansion of the neutrophil population at 24 h post-SAH, which significantly subsided by the 72 h mark (Figure 1d; Gris et al., 2019). To determine which cell type predominantly expresses the acute pathogenic program, we projected the Turquoise module score onto the UMAP embedding. Notably, the 24 h-specific injury signature was most robustly and predominantly concentrated within the neutrophil population (Figure 1e; Supplementary Figure 1e), while other immune subsets, such as microglia and macrophages, showed relatively minimal enrichment for this specific module. Functional enrichment of these neutrophils revealed pathways such as leukocyte chemotaxis, myeloid migration, and innate immune activation (Figure 1i), closely mirroring the Turquoise module’s profile (Figure 1b).

To provide spatial validation, we utilized spatial transcriptomics (ST) to map the distribution of the 24 h injury module. We identified a high topographical overlap between the Turquoise hub gene scores and the spatial spots enriched for neutrophil signatures, particularly within the perivascular and subarachnoid spaces (Figures 1g,h; Nakagawa et al., 2024). Quantitative Pearson correlation analysis further confirmed the strong spatial co-localization of the injury module and neutrophil infiltration (Figure 1j).

### N_04 is the neutrophil subpopulation most strongly enriched for the 24-h injury module

**FIGURE 2:**
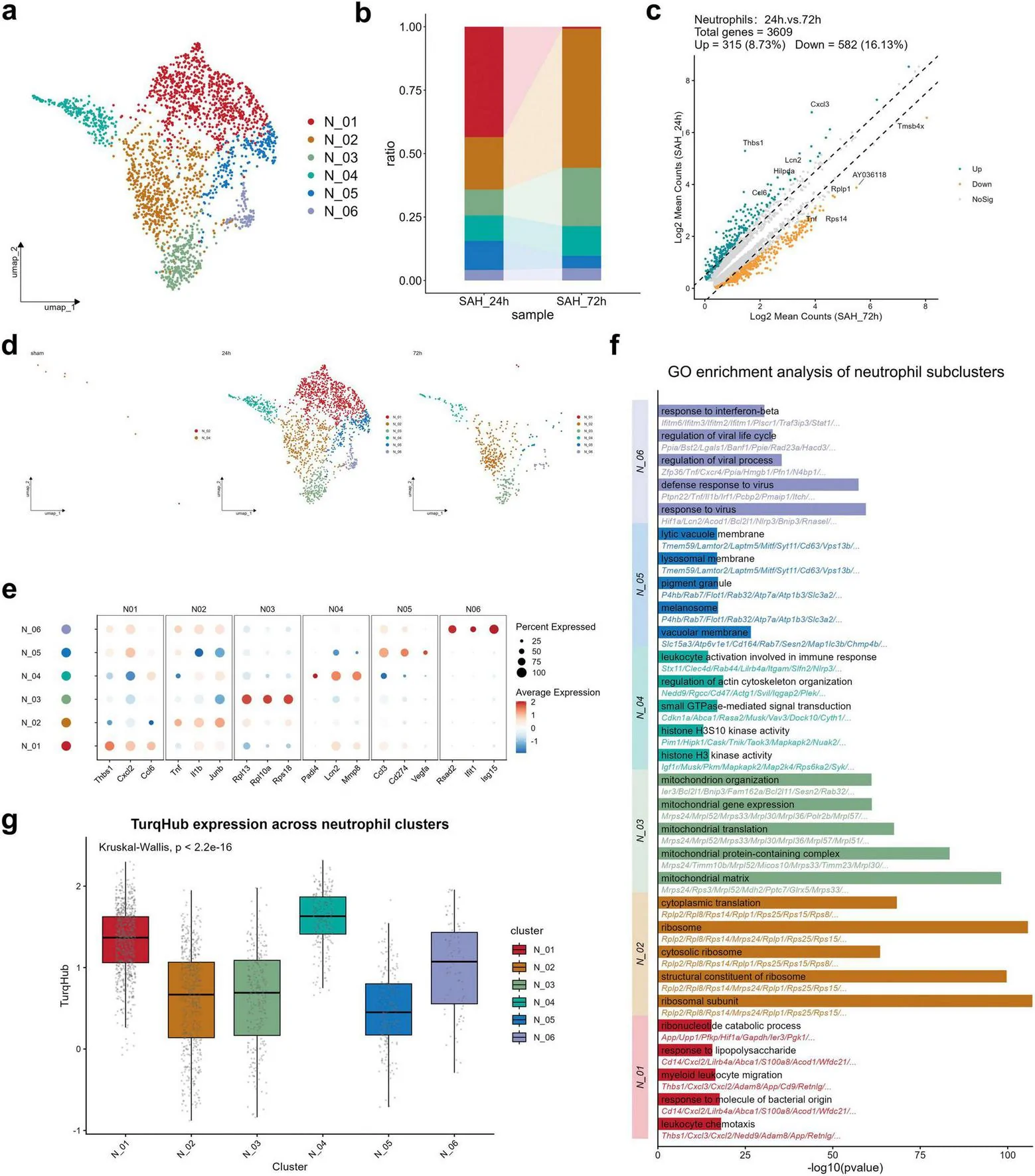
Neutrophil subclustering after SAH. **(a),** UMAP visualization of reclustered neutrophils from the integrated CD45^+^ immune-cell dataset, showing six neutrophil subclusters, N_01–N_06. **(b)** Relative abundance of each neutrophil subcluster in SAH_24 h and SAH_72 h samples. **(c)** Differentially expressed genes between neutrophils from 24 h and 72 h post-SAH samples. **(d)** UMAP visualization of neutrophil subclusters separated by Sham, 24 h and 72 h groups. **(e)** Dot plot showing representative marker genes for each neutrophil subcluster. Dot size indicates the percentage of expressing cells, and color indicates scaled average expression. **(f)** Gene Ontology enrichment analysis of marker genes from each neutrophil subcluster. **(g)** Turquoise module score across neutrophil subclusters. Statistical significance was assessed by the Kruskal-Wallis test (*n* = 2,331 cells across 5 biological samples). The boxplots display the median, interquartile range (IQR), and whiskers extending to 1.5 × IQR. *P* < 0.0001.

To resolve the internal heterogeneity of the infiltrating neutrophils, we performed high-resolution re-clustering, which partitioned the population into six distinct subclusters (N_01 to N_06) (Figures 2a,e; Chen et al., 2021). Temporal analysis revealed that these subclusters exhibit distinct distribution patterns: N_01, N_02, and N_04 dominated the acute 24 h peak, whereas N_03 and N_05 significantly expanded by 72 h (Figures 2b,d). Differential expression analysis between 24 h and 72 h further highlighted the acute activation of markers such as Thbs1, Cxcl3, and Lcn2 during the initial 24 h window (Figure 2c; Yu et al., 2021).

Gene Ontology (GO) enrichment analysis revealed highly specialized functional profiles for each subcluster (Figure 2f). N_01 was primarily associated with leukocyte chemotaxis and myeloid migration, suggesting a role in early recruitment. N_02 and N_03 appeared to be metabolically active, enriched in ribosomal translation and mitochondrial organization, respectively. N_05 was characterized by lysosomal and vacuolar membrane functions, potentially representing a phagocytic or degradative state, while N_06 exhibited a classic interferon (IFN) response signature.

Of particular importance, the N_04 subcluster appeared to represent the primary cellular substrate of the 24-h injury module. Its transcriptomic profile was uniquely characterized by the enrichment of pathways involved in leukocyte activation, actin cytoskeleton organization, and small GTPase-mediated signal transduction (Figure 2f; Mannherz et al., 2024), effectively recapitulating the functional core of the 24 h pathogenic program. This functional alignment was further corroborated by module scoring, which demonstrated that N_04 harbored the highest concentration of the Turquoise signature across all neutrophil subsets (Kruskal-Wallis, *p* < 2.2e-16) (Figure 2g). To further clarify this relationship, intersection analysis revealed a robust overlap of 67 genes between the 24-h Turquoise module and the top 500 N_04 markers (Supplementary Figure 1h), within which 5 classical NET-associated genes were identified. Collectively, these findings define N_04 as the predominant neutrophil subpopulation orchestrating the acute transcriptomic response following SAH.

### N04 exhibits a NET-competent effector program after SAH

To further elucidate the specific effector mechanisms contributing to acute post-SAH injury, we deeply mined the hub genes of the 24 h Turquoise module. This analysis revealed a prominent expression signature associated with Neutrophil Extracellular Trap (NET) formation, featuring key mediators such as Padi4, Mmp8, and Lcn2 (Supplementary Table 1; Li et al., 2010). By integrating this distinct NET-related molecular profile with our earlier GO analysis—which highlighted robust leukocyte activation and structural remodeling within the N_04 subcluster (Figure 2f)—we hypothesized that N_04 serves as the primary cellular source mediating NETosis-induced brain injury.

**FIGURE 3:**
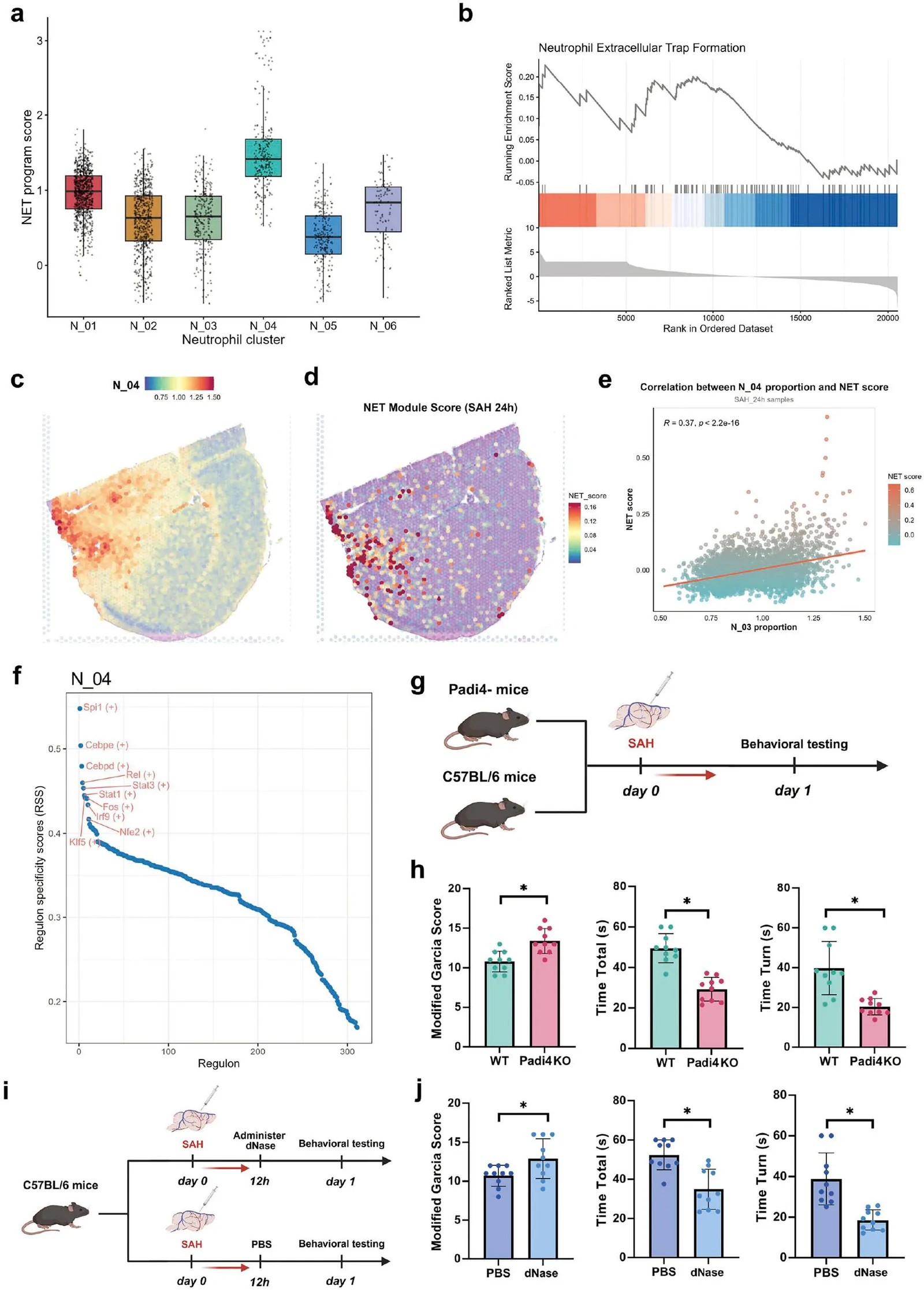
NET-associated signature of N_04 neutrophils after SAH. **(a)** Boxplot of NET scores across neutrophil subclusters, derived from a curated NET-associated gene set, with individual cells shown as dots. Statistical significance was assessed by the Kruskal-Wallis test (*n* = 2,331 cells across 5 biological samples: 8 cells from Sham, 1,922 cells from SAH 24 h, and 401 cells from SAH 72 h). The boxplots display the median, interquartile range (IQR), and whiskers extending to 1.5 × IQR. **(b)** Gene set enrichment analysis (GSEA) of the NET formation pathway in N_04 neutrophils. **(c)** Spatial distribution of the N_04 neutrophil signature in 24 h post-SAH brain sections, inferred from spatial deconvolution. **(d)** Spatial feature plot showing NET gene-set module scores in 24 h post-SAH brain sections. **(e)** Pearson correlation between N_04 abundance and NET module score in 24 h post-SAH brain sections. **(f)** SCENIC analysis identifying NET-related transcription factors enriched in N_04 neutrophils. **(g)** Behavioral testing workflow in Padi4-deficient and wild-type mice after SAH. **(h)** Behavioral assessment of Padi4-deficient and wild-type mice at 24 h post-SAH (modified Garcia score and motor function tests). **(i)** DNase I treatment workflow for NET degradation after SAH, followed by behavioral testing. **(j)** Behavioral assessment of DNase I- and vehicle-treated mice at 24 h post-SAH (modified Garcia score and motor function tests). Asterisks indicate statistically significant differences between the indicated groups (WT vs. Padi4KO in **h**; PBS vs. DNase I in **j**; *P* < 0.05).

To validate this hypothesis, we first evaluated the NETosis transcriptomic signature across the neutrophil landscape. Specifically, we calculated the NET module score based on the Neutrophil Extracellular Trap Formation pathway derived from the KEGG database (mmu04613). This gene set comprises key structural and effector genes indicative of NETosis, including representative nodes such as Mpo, Elane, Mmp8 and S100a9. Consistent with our hypothesis, N_04 exhibited a singular and significant enrichment in the NETosis score compared to all other subpopulations (Figure 3a). This finding was further substantiated by Gene Set Enrichment Analysis (GSEA), which demonstrated significant activation of the NETosis formation program within the N_04 subcluster (Figure 3b).

Subsequently, we utilized spatial transcriptomics (ST) to investigate whether this NET effector program manifests within the post-SAH brain architecture. Spatial mapping revealed a significant overlap between the N_04 signature score and the NET formation score, with both signals predominantly localized near the surgical puncture site and the ventricular borders (Figures 3c–e).

To resolve the transcriptional regulatory architecture governing this pathogenic state, we evaluated regulon specificity scores (RSS) via SCENIC analysis (Figure 3f). The top two identified regulons, Nfe2 and Cebpe (Lawrence et al., 2018), are canonical master regulators of neutrophil granulopoiesis and terminal differentiation, providing the indispensable enzymatic reservoir (e.g., granular proteins) required for NETosis. Furthermore, the remaining top-ranked regulons (including Stat1, Rel, Cebpd, Spi1, Fos, Irf1, Stat3, and Klf5) constitute a profound pro-inflammatory and stress-response network. Notably, the robust enrichment of STAT family members and Rel (an NF-κB pathway component) not only mediates intense immune activation and reactive oxygen species (ROS) bursts but also synergistically drives the transcription of critical NETosis mediators like Padi4.

Finally, we utilized *in vivo* animal models for functional validation. Compared to wild-type (WT) controls, Padi4^–^/^–^ mice exhibited significantly attenuated neurological deficits, as evidenced by higher modified Garcia scores and superior behavioral performance (Figures 3g,h). Consistently, pharmacological degradation of NETs via DNase I treatment similarly ameliorated neurobehavioral impairments (Figures 3i,j; Zeineddine et al., 2024).

### A distinct disease-associated neutrophil state with enhanced inflammatory and effector features emerges after SAH

**FIGURE 4:**
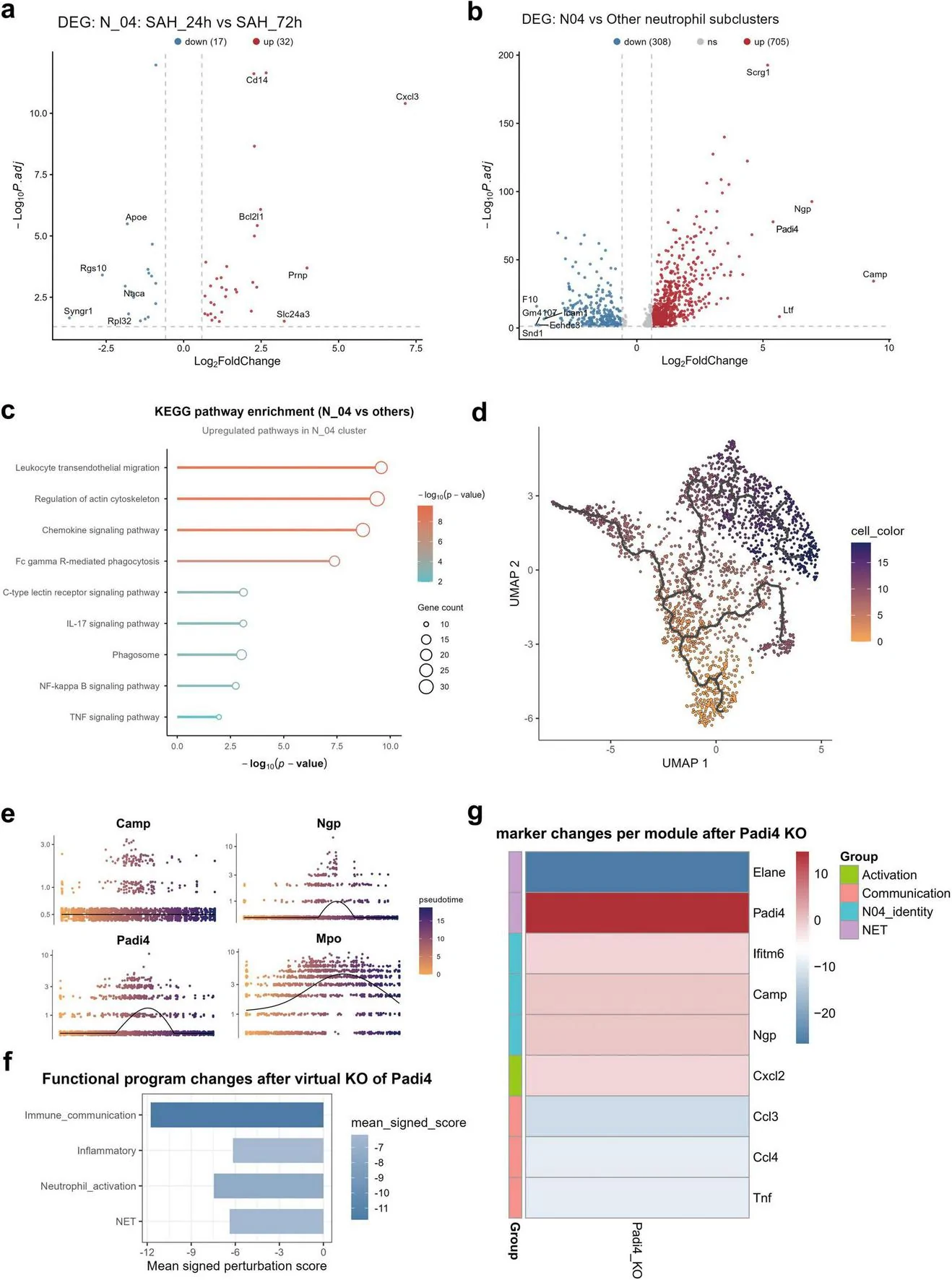
Transcriptomic dynamics and Padi4-dependent effector programs of N_04 neutrophils after SAH. **(a)** Differential gene expression in N_04 between 24 h and 72 h post-SAH, highlighting early activation and later resolution. **(b)** N_04 versus other neutrophil subclusters, showing upregulation of NETosis- and effector-related genes. **(c)** KEGG pathway enrichment of N_04 signature genes, including neutrophil activation, degranulation, and inflammatory signaling. **(d)** Pseudotime trajectory of neutrophils, with N_04 representing a terminal effector state. **(e)** Expression dynamics (normalized expression values) of representative N_04 effectors along pseudotime. **(f)** Functional module perturbation after *in silico* Padi4 knockout, showing downregulation of NETosis, activation, and inflammatory programs. **(g)** Gene-level perturbation heatmap, highlighting suppression of NETosis mediators and N_04 identity markers.

To further define the molecular identity of the N_04 subcluster, we conducted a comprehensive transcriptomic analysis across neutrophil populations. Differential gene expression (DEG) analysis revealed that N_04 is characterized by the robust upregulation of effector-related genes, including Padi4, Scrg1, Ngp, Camp, and Ltf, while genes involved in cell adhesion and basal signaling, such as Icam1, F10, and Snd1, were significantly downregulated (Figure 4b; Xie et al., 2020). Functional enrichment via KEGG pathways confirmed that these upregulated signatures are predominantly associated with neutrophil activation and degranulation pathways (Figure 4c).

The N_04 state exhibited a sharp temporal transition between the acute (24 h) and subacute (72 h) phases of SAH. At 24 h, N_04 was enriched with pro-inflammatory and recruitment-related markers, such as Cd14, Cxcl3, Bcl2l1, Prnp, and Slc24a3. By 72 h, the expression of these genes declined, accompanied by the emergence of homeostatic or regulatory markers, including Apoe, Rgs10, Naca, Syngr1, and Rpl32 (Figure 4a; Silvestre-Roig et al., 2019).

To resolve the developmental origin of this state, we performed pseudotime trajectory analysis. Setting N_03 as the root due to its enrichment in baseline ribosomal and mitochondrial signatures, the trajectory progressed through N_02 before diverging into three distinct lineages: one branch terminating at N_04, a second proceeding through N_05 toward N_06, and a third routing from N_02 through N_01 toward N_05 (Figure 4d). Notably, the N_04 branch represented a terminal effector fate, characterized by the continuous and significant upregulation of key driver genes such as Padi4, Camp, Ltf, and Ngp (Figure 4e).

To explore the potential regulatory role of Padi4 in sustaining this pathogenic state, we performed an *in silico* perturbation analysis using the characteristic marker genes of the N_04 subcluster. While this provides hypothesis-supporting evidence rather than definitive *in vivo* proof, the simulated deletion of Padi4 predicted a significant downstream suppression (indicated by negative signed perturbation scores) across four core functional modules within the N_04 transcriptome: neutrophil activation, NETosis, stress response, and degranulation (Figure 4f). Importantly, these modules were independently pre-defined based on existing literature and the KEGG database prior to the analysis. Gene-level analysis further confirmed the robust predicted downregulation of specific downstream targets within these modules, particularly key secondary granule components (Figure 4g).

### Intercellular communication analysis reveals N_04 neutrophils as a primary signaling receiver after SAH

**FIGURE 5:**
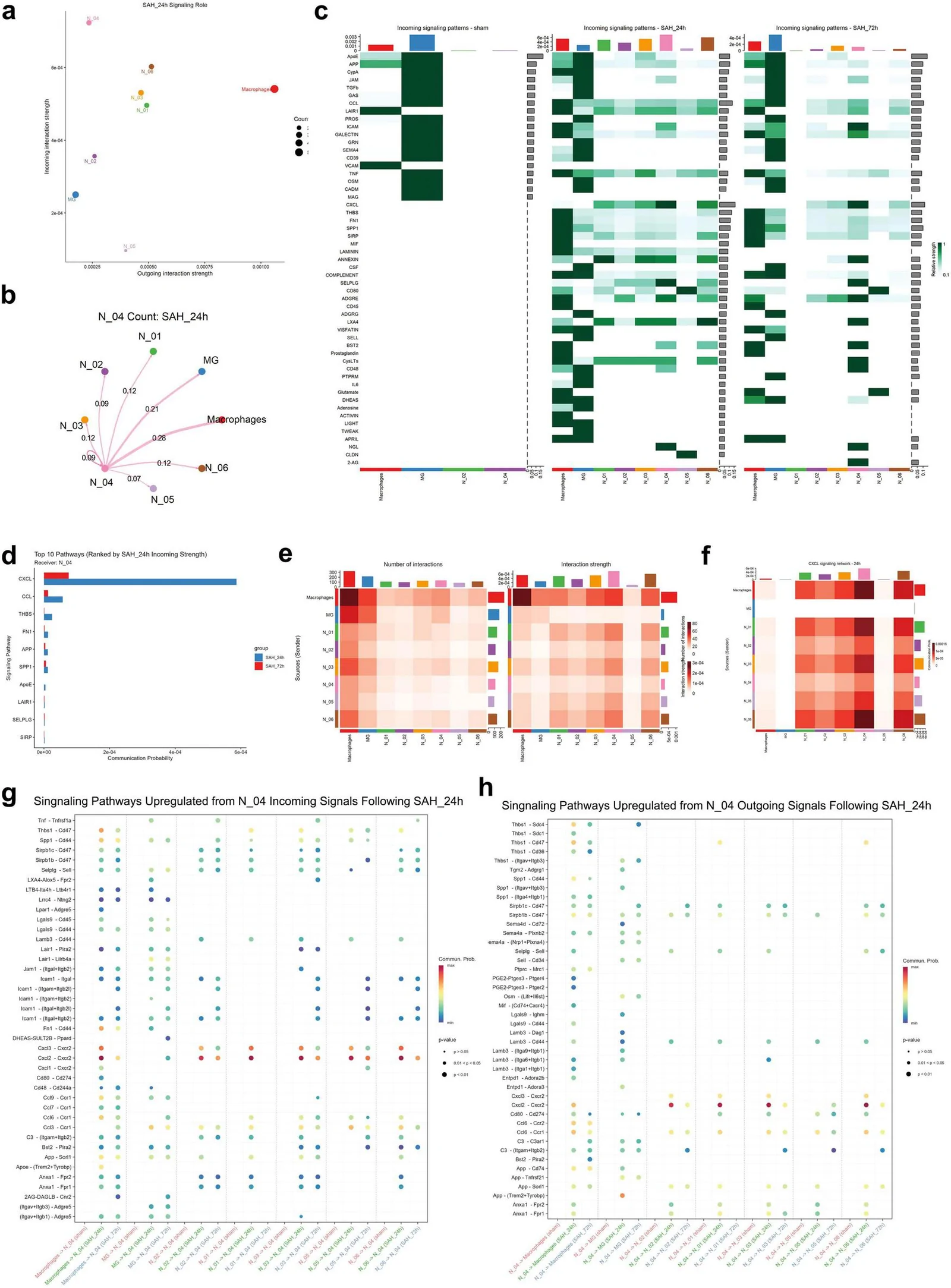
Intercellular communication networks identify N_04 as a primary signaling receiver after SAH. **(a)** Signaling role analysis of immune subsets at 24 h post-SAH, showing outgoing and incoming interaction strengths. **(b)** Circle plots showing outgoing interaction counts from N_04 to other immune populations at 24 h post-SAH. **(c)** Heatmap illustrating incoming signaling patterns (based on communication probabilities) of macrophage, microglial, and neutrophil subpopulations. **(d)** Top 10 incoming signaling pathways from N_04 ranked by communication probability in SAH_24 h and compared with SAH_72 h. **(e)** Heatmaps of interaction number and interaction strength among immune subsets at 24 h post-SAH. **(f)** Heatmaps of CXCL signaling networks at 24 h post-SAH. **(g)** Bubble plot showing ligand–receptor pairs upregulated in N_04 incoming signaling at 24 h post-SAH. **(h)** Bubble plot showing ligand–receptor pairs upregulated in N_04 outgoing signaling at 24 h post-SAH.

To elucidate the mechanisms driving N_04 neutrophil activation early after SAH, we inferred cell-cell communication networks using CellChat. (Jin et al., 2021). Network centrality analysis at 24 h post-SAH revealed that N_04 predominantly acted as a major signaling receiver, exhibiting the highest incoming interaction strength among immune subsets (Figure 5a). Furthermore, interaction counts highlighted extensive crosstalk, with the most robust communication occurring between N_04 and macrophages (Figure 5b).

Mapping global incoming signaling patterns showed that N_04 neutrophils were significantly enriched for incoming CXCL, SELPLG, and NGL signals at 24 h post-SAH (Figure 5c). Notably, among the top 10 pathways at this acute injury time point, the CXCL pathway exhibited the strongest communication probability (Figure 5d), making it our primary focus.

Detailed network analysis revealed N_04 as the primary target for signals from macrophages and the N_01, N_03, and N_06 neutrophil subclusters (Figure 5e). This receiver-centric dynamic was strictly conserved within the CXCL-specific network, where these same populations served as the dominant sources of CXCL ligands targeting N_04 (Figure 5f). At the ligand–receptor level, incoming CXCL signaling to N_04 was predominantly driven by Cxcl2-Cxcr2 and Cxcl3-Cxcr2 interactions (Figure 5g). Concurrently, N_04 maintained a specific outgoing profile, directing Cxcl2-Cxcr2 signals back toward the N_01, N_02, N_03, and N_06 subclusters (Figure 5h). These findings highlight an early post-SAH inflammatory paradigm driven by macrophage-neutrophil chemotactic crosstalk and a self-amplifying auto-chemotactic loop among neutrophils.

## Discussion

The limitations of broad-spectrum immunomodulatory therapies in improving clinical outcomes after subarachnoid hemorrhage (SAH) (Pfnür et al., 2025; Quandt et al., 1982) underscore a profound gap in our understanding of the acute neuroimmune ecosystem (Caner et al., 2012). Historically, post-SAH early brain injury (EBI) has been viewed through the lens of bulk tissue inflammation, which obscures the highly compartmentalized and dynamic nature of the infiltrating immune compartment (Robba et al., 2024). In this study, we identify a transient, highly pathogenic temporal window peaking at 24 h post-ictus, which is not driven by pan-immune activation, but rather orchestrated by a specific, terminally differentiated neutrophil subpopulation (N_04). By defining its pathogenic reliance on PADI4-dependent NETosis and its role as a core intercellular signaling hub, our findings indicate that EBI is closely associated with a cell-state-specific pathological program (Zeineddine et al., 2024). Compared to the traditional view of a generalized inflammatory cascade, this provides a more refined and precise on the acute microenvironment.

Traditionally, neutrophils infiltrating the injured central nervous system (CNS) have been considered a homogenous, short-lived population of generic effectors primarily tasked with debris clearance (Kolaczkowska and Kubes, 2013). However, our single-cell trajectory and spatial analyses reveal their profound transcriptional heterogeneity and specialized developmental fates (Xie et al., 2020). The emergence of the N_04 state challenges the classic “bystander” view of neutrophils in SAH. Uniquely localized to the modeling puncture site and ventricular margins, N_04 represents a terminal effector fate distinct from earlier recruitment (N_01) or subacute regulatory (N_05) states. This rapid, highly specific polarization within the first 24 h suggests that the local hemorrhagic microenvironment—likely rich in damage-associated molecular patterns (DAMPs) and breakdown products—actively imprints a hyper-inflammatory phenotype onto migrating myeloid cells, which may drive them toward a tissue-destructive trajectory (Zhu et al., 2025). Notably, this rapid expansion at 24 h and decline by 72 h closely mirrors the classical acute phase response. This suggests that the N_04 state represents a fast, time-limited inflammatory burst in the injured brain that naturally resolves as the acute phase ends.

A central mechanistic advance of our study is the characterization of the molecular machinery governing N_04 pathogenicity. While the role of Neutrophil Extracellular Traps (NETs) is well-documented in infectious diseases, their aberrant deployment in sterile CNS injury remains an area of active investigation (Brinkmann et al., 2004). Our SCENIC analysis reveals that N_04 is hardwired for NETosis through a dense regulatory network governed by Nfe2, Cebpe, and robust NF-κB/STAT signaling. Strikingly (Xu et al., 2025), our *in silico* perturbation modeling demonstrated that the deletion of a single node—Padi4—precipitates a catastrophic collapse of the N_04 effector modules, including degranulation and stress responses. This computational prediction was supported by our *in vivo* functional data, where both the genetic ablation of Padi4 and the pharmacological degradation of NETs profoundly rescued acute neurobehavioral deficits (Zeineddine et al., 2024). Collectively, this continuous chain of evidence—spanning the molecular upregulation of Padi4/Mmp8, transcriptomic enrichment for NETosis pathways, epigenetic hardwiring via Nfe2/Cebpe, and functional rescue in vivo—strongly supports the characterization of N_04 as a highly specialized “NET-competent” state rather than merely a broadly activated neutrophil cluster. This consolidated framework provides compelling evidence that the PADI4-NETosis axis not just as a byproduct of general inflammation, but as a non-redundant, primary driver of acute post-SAH brain injury.

Beyond its direct neurotoxic capabilities via chromatin extrusion and protease release, our intercellular communication modeling positions the N_04 state as a central signaling receiver within the early neuroimmune niche. Rather than acting solely as an independent orchestrator, N_04 is deeply integrated into, and driven by, reciprocal crosstalk with the surrounding inflammatory microenvironment. Specifically, we identified a robust CXCL-mediated chemotactic network where N_04 serves as the primary target for signals derived from macrophages and other neutrophil subclusters. (Lämmermann et al., 2013), This intense incoming signaling is coupled with a self-amplifying CXCL autocrine/paracrine loop among neutrophils, which likely sustains persistent neutrophil swarming and activation at the injury site.

Our findings may have potential significant translational implications. The distinct spatiotemporal restriction of the N_04 state (peaking at 24 h, declining by 72 h) provides a potential therapeutic window. Interventions aimed at broad immune suppression often fail due to the indiscriminate disruption of later-stage reparative processes (such as those potentially mediated by N_05 or homeostatic microglia) (Robba et al., 2024). Instead, highlight the potential value of “precision immunomodulation”: selectively targeting the N_04 state or its primary weapon (PADI4-dependent NETosis) within the hyper-acute phase. Such an approach could dismantle the primary engine of EBI without compromising the necessary debris clearance and tissue remodeling required for long-term recovery.

Despite these advances, several limitations must be acknowledged. First, while our *in silico* and murine models suggest a prominent pathogenic role for N_04, cross-species validation using human post-SAH cerebrospinal fluid (CSF) (Schafflick et al., 2020) or brain tissue is imperative to confirm the presence and kinetic profile of an analogous NET-competent neutrophil state in patients. Second, although we mapped the robust transcriptional signature of NETosis, integrating high-resolution spatial proteomics would provide definitive validation of localized granular protein deposition (e.g., MMP8, LCN2) within the intact brain architecture. The precise upstream DAMPs within the hemorrhagic niche that trigger the divergence of the N_04 trajectory require further functional deconvolution. Third, the limited sample size in our transcriptomic analyses may restrict the capture of inter-individual heterogeneity. Thus, large-cohort validation is needed to confirm the broader generalizability of these findings.

In conclusion, by integrating single-cell and spatial transcriptomics with computational modeling, we have characterized the acute immune architecture of SAH. Our findings highlight the NET-competent N_04 neutrophil subset as a spatiotemporally restricted, critical instigator of early brain injury. These insights provide a high-resolution cellular and molecular framework that paves the way for the next generation of targeted, cell-state-specific therapies for subarachnoid hemorrhage.

## Materials and methods

### Experimental model and *in vivo* assays

#### Ethical approval

All animal procedures were approved by the second affiliated hospital of Zhejiang university - Animal Ethics Committee (Approval no. AIRB-2024-0285) and were performed according to the Guiding Principles for the Care and Use of Laboratory Animals Approved by Animal Regulations of National Science and Technology Committee of China.

#### Animals

Adult male C57BL/6J wild-type (WT) mice (8–10 weeks old, body weight 22–26 g) were purchased from Shanghai SLAC Laboratory Animal Co., Ltd., (Shanghai, China). Padi4-deficient (Padi4-/-) mice on a C57BL/6 background were purchased from GemPharmatech Co., Ltd., (Jiangsu, China). Age- and weight-matched WT C57BL/6J mice were used as controls for the Padi4-/- cohorts. All mice were housed in a specific pathogen-free (SPF) facility under a temperature-controlled environment (22 ± 2 °C) and humidity (50 ± 10%) with a standard 12/12-h light/dark cycle. Standard rodent chow and sterilized tap water were provided *ad libitum*. Animals were allowed to acclimate to the environment for at least 2 weeks prior to any experimental procedures. Animals were randomly assigned to their respective experimental groups. To minimize bias, investigators were blinded to the group allocations and genotypes during all surgical procedures, behavioral outcome assessments, and data analyses.

#### Mouse SAH model

Experimental SAH was induced by endovascular filament perforation as described previously (Chen et al., 2023). Mice were anesthetized with 4% isoflurane in 30% oxygen for induction, followed by endotracheal intubation and mechanical ventilation (MiniVent 845, Hugo Sachs Elektronik/Harvard Apparatus). Carprofen (4 mg/kg, s.c.) was given pre-emptively; anesthesia was maintained with an intraperitoneal mixture of fentanyl (0.05 mg/kg), midazolam (5 mg/kg), and medetomidine (0.5 mg/kg). Body temperature was maintained at 37°C using a feedback-controlled heating pad and rectal probe. Through a midline cervical incision, the common carotid artery (CCA), internal carotid artery (ICA), and external carotid artery (ECA) were exposed and partially mobilized. The ECA was ligated distally, and a 5-0 prolene monofilament (12 mm, tip rounded and blunted) was introduced via the ECA stump and advanced into the ICA. Perforation of the anterior cerebral artery at the Circle of Willis was achieved by gently advancement of the filament. A sudden rise in intracranial pressure with a concomitant drop in regional cerebral blood flow signaled successful perforation, after which the filament was immediately withdrawn and the ECA ligated. Sham animals underwent identical exposure without perforation. At the end of surgery, anesthesia was antagonized per protocol to allow rapid recovery. Mice were maintained on a warming pad until fully awake and received carprofen (4 mg/kg, s.c., every 24 h for 2–3 days). Postoperative care included monitoring for neurological deficits, weight loss, and hydration status.

#### Neurological scores and behavioral testing

Neurological scores and all behavioral assessments for each group were conducted at a similar and relatively fixed time during daylight hours. To minimize bias, an independent investigator blinded to the experimental groups performed all evaluations at 24 h after subarachnoid hemorrhage (SAH). For the pole and wire hanging tests, mice were trained three times daily for 3 days before the formal testing began.

Modified Garcia score: Short-term neurological function was evaluated using the Modified Garcia scoring system. This assessment comprises six sub-tests: spontaneous activity, symmetry of limb movement, forepaw outstretching, climbing, body proprioception, and response to vibrissae touch. Each item is scored from 0 to 3, yielding a total score ranging from 3 to 18. A higher score represents better neurological function.

Pole test: Animals were placed head-up on top of a vertical pole (50 to 55 cm in height, 8 to 10 mm in diameter) and trained to turn around and descend. Scoring began when the animal initiated the turning movement. The time taken to make a complete 180° turn (T_turn) and the total latency to reach the ground (T_total) were recorded. If a mouse descended laterally without turning, T_total was also attributed to T_turn. If a mouse made a turn, descended halfway, and fell, the recorded time stopped upon hitting the floor. The maximum observation time was set to 60 s.

Wire hanging test: This test was conducted to evaluate grip strength, balance, and motor endurance. Mice were trained to suspend their bodies by holding onto a single wire stretched between two posts positioned 50 to 60 cm above the ground. To ensure the assessment focused on forelimb strength and prevent the use of all four paws, the hind limbs were gently covered with adhesive tape. A pillow was placed beneath the wire to prevent injury upon falling. The primary endpoint for motor performance was the “latency to fall,” with the maximum testing time capped at 60 s.

#### *In vivo* DNase I treatment

To investigate the therapeutic efficacy of degrading neutrophil extracellular traps (NETs) following subarachnoid hemorrhage, mice were treated with recombinant Deoxyribonuclease I (DNase I, RNase-free; Beyotime Biotechnology, Shanghai, China; Cat# D7073). In accordance with the manufacturer’s guidelines to preserve optimal enzymatic activity, the DNase I was maintained on ice during preparation and strictly protected from repeated freeze-thaw cycles. The enzyme was freshly diluted in sterile 0.9% normal saline, which served as the vehicle, immediately prior to use. At 12 h after the induction of SAH, mice randomly assigned to the treatment group were administered DNase I via intraperitoneal injection at a dosage of 5 mg/kg. The vehicle control group received an equivalent volume of sterile 0.9% normal saline at the identical time point. The treatment was administered as a single dose to systematically degrade circulating and tissue-deposited NETs. To minimize bias, investigators were blinded to the treatment allocations during all subsequent neurobehavioral, histological, and molecular outcome evaluations.

### Tissue processing and library sequencing

#### Tissue collection and processing

At designated experimental endpoints, mice were anesthetized with an intraperitoneal overdose of pentobarbital (100 mg/kg). Following the loss of righting and pedal reflexes, the animals were euthanized via transcardial perfusion with ice-cold PBS to minimize blood contamination. Subsequently, the ipsilateral (injured) cerebral hemisphere was carefully dissected on a pre-chilled surface.”. The meninges, olfactory bulb, and cerebellum were removed to ensure consistency across samples. For scRNA-seq, animals were transcardially perfused with ice-cold PBS to minimize blood contamination, and hemispheres were processed immediately for cell dissociation. Fresh tissue was enzymatically and mechanically dissociated using the Adult Brain Dissociation Kit (Miltenyi Biotec, type T) in combination with a gentleMACS Octo Dissociator with heaters, following the manufacturer’s protocol. Single-cell suspensions were filtered through a 70 μm cell strainer and separated on a 30%/70% Percoll discontinuous gradient (800 g (+3 −1), 60 min, 4 °C, no brake). The immune cell layer at the 30/70% interphase was collected and washed (HBSS + 0.5% BSA). Only samples with > 85% cell viability (assessed by trypan blue exclusion) were advanced to library preparation. For ST, brains were embedded in optimal cutting temperature (OCT) compound, rapidly snap-frozen in pre-chilled isopentane on dry ice, and stored at –80 °C until cryosectioning.

#### ScRNA-seq–library preparation and sequencing

Single-cell barcoding and library construction were performed using the Chromium Single Cell 3’ v2 platform (10x Genomics). Libraries were sequenced on an Illumina NovaSeq 6000 system, targeting a depth of ≥ 100,000 reads per cell using a paired-end 120 bp configuration.

#### ST–library preparation and sequencing

Brains were cryosectioned at 10 μm and mounted onto Visium Spatial Gene Expression slides (10x Genomics). Sections were fixed, H&E stained, and imaged prior to permeabilization. Library construction followed the Visium Spatial Gene Expression User Guide (CG000239). Final libraries were sequenced on an Illumina NovaSeq 6000 platform with a paired-end 150 bp configuration, targeting a depth of ≥ 100,000 reads per spot.

### Single-cell and spatial transcriptomic bioinformatics

#### DEG analysis

All differential expression analyses were performed in Seurat v5.3.0 using the two-side Wilcoxon rank-sum test, with *P* values adjusted by the Benjamini–Hochberg (BH) method. Unless noted otherwise, significance thresholds were log2FC > 0.25, gene detected in ≥ 10% of cells in the tested group, and BH-adjusted *P* < 0.05. For cluster-level markers, each cluster was compared against all remaining cells (Seurat FindAllMarkers, only.pos = TRUE) and genes were ranked by effect size (log2FC), from which representative top markers were reported. For pairwise group contrasts (e.g., sham vs SAH_24 h; SAH_24 h vs. SAH_72 h), DEGs were identified with FindMarkers under the same thresholds, retaining both up and downregulated genes; the resulting gene sets were used for downstream functional enrichment.

#### ScRNA-seq data preprocessing and quality control

All scRNA-seq data preprocessing and quality control (QC) were performed in R v4.5.1 with Seurat package v5.3.0 (Butler et al., 2018; Hao et al., 2021; Satija et al., 2015; Stuart et al., 2019). Raw FASTQ files were processed with CellRanger (10x Genomics) to generate gene–cell matrices, which were imported via Read10X and initialized with CreateSeuratObject (default thresholds min.cells = 3, min.features = 200) (Zhu et al., 2025). Per cell mitochondrial fraction was computed with PercentageFeatureSet (pattern ^mt-), and cells were filtered by:nFeature_RNA with < 200 or > 9,000 percent.mt > 10% and abnormally low/high nCount_RNA (flagged by FeatureScatter) to exclude low-quality cells and putative doublets. After filtering, counts were normalized with NormalizeData (LogNormalize, scale. factor = 1e4), and ScaleData was used to regress unwanted variation (e.g., nCount_RNA, percent.mt). Highly variable genes were selected with FindVariableFeatures, and dimensionality reduction was performed with RunPCA based on the top variable features. QC metrics (nFeature_RNA, nCount_RNA, and percent.mt) were visualized using VlnPlot and FeatureScatter.

#### Cell clustering, annotation, and doublet removal

Batch effect correction across samples was corrected with Harmony v1.2.3 by applying RunHarmony to the PCA embeddings and using orig.ident as the grouping variable, thereby aligning cells across biological replicates while preserving biological variation (Zhu et al., 2025). After integration, we built a shared nearest neighbor (SNN) graph with FindNeighbors and performed unsupervised clustering using FindClusters; the resolution was tuned empirically to maximize separation of biologically meaningful populations while avoiding over-clustering. Two-dimensional embeddings for visualization were generated with RunUMAP on the Harmony-corrected space.

Cell-type annotation combined three complementary approaches: (i) automated labeling with SingleR against reference datasets; (ii) consultation of the CellMarker database for curated marker; and (iii) manual curation using canonical lineage marker from the literature. To minimize false positives, putative doublets and low-quality clusters were removed based on UMAP inspection and co-expression of mutually exclusive lineage markers (e.g., lymphoid vs myeloid signatures); clusters dominated by mitochondrial/ribosomal gene expression and lacking clear biology were also excluded.

DEGs were identified with Seurat FindMarkers function in (Wilcoxon rank sum test, min.pct = 0.25, logfc.threshold = 0.25), and resulting signatures were cross-validated against published references to confirm assignments.

#### Gene set enrichment analysis–module scoring

Gene set were obtained from MSigDB (version 2025.1, accessed in November 2025), focusing on Gene Ontology Biological Processes (GOBP) terms. Gene sets were downloaded in GMT format, and when acquired, human-to-mouse conversion was performed with msigdbr R package. Additionally, the specific gene signature for Neutrophil Extracellular Trap (NET) formation was directly retrieved from the KEGG database (Pathway: mmu04613). The full list of gene sets used is provided in Supplementary Table 2.

Module scores were computed in Seurat v5.3.0 using AddModuleScore (single cells) and per-spot for spatial data. For each cell/spot, the score equals the average expression of genes in the target set minus the average expression of matched control gene sets generated by Seurat, yielding a standardized enrichment estimate.

For scRNA-seq, enrichment scores were visualized using VlnPlot (clusters-wise distribution) and FeaturePlot (UMAP overlays). For ST, enrichment scores were displayed with SpatialFeaturePlot to map pathway distribution across brain sections; for a given program, color scales were harmonized across samples/time points to enable direct comparison.

#### Functional enrichment (GO/KEGG) and gene set enrichment analysis (GSEA)

Functional enrichment analyses were performed in R v4.5.1 using clusterProfiler package v4.16.0 (Wu T. et al., 2021) with Mus musculus as the reference background. For GO enrichment, DEGs lists were analyzed with compareCluster/enrichGO (ontology = “All”); *P* values were adjusted by Benjamini–Hochberg, and terms with adjusted *P* < 0.05 and *q* < 0.05 were considered significant. For KEGG enrichment, DEGs were split into upregulated and downregulated sets and assessed separately with enrichKEGG (organism = “mmu”, pAdjustMethod = “fdr”); significant pathways were exported for downstream visualization, and –log10(*P*) values were plotted as positive (upregulated) or negative (downregulated) bars in ggplot2 to indicate directionality. In addition, classic GSEA (clusterProfiler) was performed by ranking genes by avg_log2FC and tested against KEGG pathways and MSigDB (v2025.1); significance was based on normalized enrichment scores (NES) and FDR q-values, with FDR < 0.25 considered enriched.

#### High-dimensional weighted gene co-expression network analysis (hdWGCNA)

To identify co-expressed gene modules associated with specific temporal injury states and immune cell populations after SAH, we performed network analysis using the hdWGCNA (v0.4.10) framework. Given the inherent sparsity of single-cell transcriptomic data, we first aggregated transcriptionally similar cells into metacells (*k* = 25 nearest neighbors) grouping by sample origin and Seurat cluster identities. The metacell expression matrix was normalized and restricted to highly variable genes to construct the co-expression network.

To ensure a scale-free topology, a soft-thresholding power of 5 was empirically selected based on scale-free topology model fit. Network construction and consensus topological overlap matrix (TOM) calculation were subsequently performed. Module eigengenes (MEs), representing the first principal component of the expression matrix for each module, were computed to summarize module-level expression profiles. Module connectivity was quantified to rank gene importance, identifying the top hub genes (ranked by weighted degree) within biologically relevant modules (e.g., the 24 h post-SAH associated Turquoise module).

To evaluate module-trait associations, ME scores were averaged across annotated cell types and sample time points (Sham, 24 h, and 72 h post-SAH) and visualized using hierarchical clustering heatmaps (pheatmap). Furthermore, to project module activity back to the single-cell and spatial resolution, the top 100 hub genes of the Turquoise module were scored using the AddModuleScore function in Seurat, allowing for the visualization of hub module activities across different sub-populations and spatial spots.

#### Cell-cell communication analysis

Cell-cell communication networks across the Sham, SAH 24 h, and SAH 72 h groups were inferred using the R package CellChat (v2.2.0) and the CellChatDB.mouse database. Normalized expression matrices and cell annotations were extracted to create independent CellChat objects for each group. We identified overexpressed ligand-receptor interactions and computed their communication probabilities based on average gene expression using the computeCommunProb function. To ensure robustness, calculations were not weighted by cell population size (population.size = FALSE), and interactions involving fewer than 10 cells were excluded. Pathway-level communication probabilities were then calculated (computeCommunProbPathway), followed by network aggregation (aggregateNet) and network centrality analysis to define major signaling roles (senders, receivers, mediators, and influencers), with a specific focus on the N_04 neutrophil subset. Finally, the CellChat objects were merged (mergeCellChat) to quantitatively compare the interaction numbers, strengths, and dynamic alterations of specific signaling pathways across disease progression.

#### Trajectory and pseudotime analysis

We reconstructed differentiation dynamics using the trajectory inference framework Monocle3 to establish lineage topology and pseudotime ordering.

For Monocle3 (v1.4.26) (Qiu et al., 2017a,2017b; Trapnell et al., 2014), Seurat objects for macrophage and neutrophil subclusters were converted into cell_data_set objects. Expression matrices, cell metadata (including cluster labels), and gene annotations were transferred directly from Seurat. Dimensionality reduction used preprocess_cds; UMAP embeddings computed in Seurat were imported to maintain visualization consistency. Trajectory graphs were learned with learn_graph (minimal branch length = 10). Root nodes were assigned based on biologically motivated starting clusters, and pseudotime was computed with order_cells. Genes varying along pseudotime were identified using Moran’s I (graph_test), and cells were visualized in UMAP space colored by pseudotime.

The resulting Monocle3 analysis delineated clear developmental hierarchies and pseudotime orderings, establishing the inferred differentiation trajectories.

#### Gene regulatory network (GRN) inference

Based on the single-cell gene expression matrix and TFs expression status, a co-expression module between TFs and candidate target genes was constructed using the pySHENIC software package (v.0.11.2) (Van de Sande et al., 2020). RcisTarget software was used to identify modules with significantly enriched regulatory factor binding motifs (Motif) in target genes, and create regulatory network modules (Regulons) containing TF and directly targeted genes. “AUCell” package was used to score the activity of each group of regulators in each single cell. Subsequently, a heatmap of normalized regulon AUC was generated using R.

#### ST analysis

Raw ST data were processed with Space Ranger to generate filtered_feature_bc_matrix.h5 outputs (Hao et al., 2024), then imported into Seurat via Load10X_Spatial. To reduce batch effects across time points, data were normalized with SCTransform (assay = “Spatial”), followed by PCA and integration with Harmony (RunHarmony, grouping by orig.ident). Harmony-corrected embeddings were used for all downstream visualization and analyses.

Spatial Pathway activity (module scoring) was quantified on the merged ST object using Seurat AddModuleScore. Gene sets were primarily drawn from the MSigDB GOBP collection (version 2025.1, accessed March 2025), the complete list of the gene sets used is provided in Supplementary Table 2. For any given program, color scales were harmonized across sections/time points to allow direct comparison.

Cellular composition per spot was inferred using RCTD (R package spacexr, v2.2.1), with the matched scRNA-seq dataset as reference (raw count matrix, cell cluster annotations, and per-cell UMI counts assembled via Reference). For each section (sham, SAH-24 h, SAH-72 h), counts and tissue coordinates were extracted from the Seurat object to construct a SpatialRNA object (GetTissueCoordinates), create.RCTD and run.RCTD were executed in “full” mode, and spot-level deconvolution weights were appended to Seurat metadata. Primary and secondary cell-type weights were visualized with SpatialDimPlot and SpatialFeaturePlot.

#### *In silico* gene perturbation and module analysis

To investigate the transcriptomic and regulatory consequences of Padi4 deletion within the neutrophil N_04 subpopulation, we performed an *in silico* knockout analysis using the scTenifoldKnk R package (version 1.0.3). Briefly, a single-cell gene regulatory network (scGRN) was constructed using Padi4 and the top 2000 highly variable genes (identified via Seurat’s “vst” method). scTenifoldKnk simulated the knockout by setting Padi4 expression to zero, utilizing machine learning-based tensor network alignment to quantify systemic structural perturbations on downstream genes. The resulting perturbation magnitude and direction for each gene were then ranked by test statistics (e.g., Z-score and log2 fold-change).

To evaluate the systemic functional impact, we assessed transcriptomic shifts across four predefined biological programs: immune communication, inflammatory response, neutrophil activation, and NET formation, with gene sets sourced from MSigDB (version 2025.1, accessed in November 2025). Module-level perturbations were quantified by calculating the mean signed and absolute perturbation scores of their constituent genes. Finally, top perturbed genes, functional module shifts, and key marker dynamics were visualized using bar plots and heatmaps.

### Quantification and statistical analysis

#### Reproducibility and software

All analyses were conducted in R 4.5.1. Core single-cell and spatial workflows used Seurat v5.3.0 (with SeuratObject v5.1.0, sctransform v0.4.2, Matrix v1.7-3) and integration with harmony 1.2.3; data containers relied on SingleCellExperiment v1.30.1 and SummarizedExperiment v1.38.1. Clustering and visualization used ggplot2 v3.5.2, patchwork v1.3.1, cowplot v1.2.0, and ComplexHeatmap v2.24.1. Functional enrichment and GSEA employed clusterProfiler v4.16.0, DOSE v4.2.0, enrichplot v1.28.4, fgsea v1.34.2, KEGGREST v1.48.1, GSEABase v1.70.0 and org.Mm.eg.db v3.21.0.Trajectory inference used monocle3 v1.4.26; differentiation potency was estimated with CytoTRACE2 v1.1.0. GRN analyses followed the SCENIC framework (GENIE3 v1.30.0, RcisTarget v1.28.1, AUCell v1.30.1, SCENIC (v1.3.1). Spatial deconvolution was performed with spacexr/RCTD v2.2.1.Weighted gene co-expression network analysis was conducted using hdWGCNA v0.4.10 and WGCNA v1.74.Cell-cell communication networks were inferred using CellChat v2.2.0. *In silico* gene knockout simulations and perturbation analyses were performed using scTenifoldKnk v1.0.3.

### Statistical analysis

Statistical analyses were conducted using IBM SPSS Statistics or GraphPad Prism 9 software. Data are presented as the mean ± standard error of the mean (SEM). For comparisons between two independent groups, a two-tailed unpaired Student’s *t*-test (parametric) or a Mann–Whitney U test (nonparametric) was employed. For comparisons involving three or more groups, a one-way analysis of variance (ANOVA) followed by Tukey’s or Dunnett’s *post hoc* tests was utilized. A *P*-value < 0.05 was considered statistically significant. No sample outliers were excluded from the analyses. All animal neurobehavioral experiments were independently repeated at least three times with consistent results.

## Data Availability

All sequencing datasets analyzed in this study, including both scRNA-seq and spatial transcriptomics data, are available in the GEO database under accession codes GSE311852 and GSE311853.

## Supplementary material

Supplementary Figure 1 WGCNA parameter selection, module validation and temporal immune-cell landscape after SAH. **(a)** Scale-free topology analysis used to determine the soft-thresholding power for WGCNA of integrated CD45^+^ immune-cell transcriptomes. The selected power for network construction is indicated. **(b)** Gene dendrogram generated by hierarchical clustering of co-expressed genes, with colored bars denoting WGCNA module assignments. **(c)** Correlation between module membership and gene significance within the Turquoise module, supporting its association with the 24 h post-SAH injury state. **(d)** Gene Ontology enrichment analysis of Yellow module genes. Dot size indicates gene count, and color represents enrichment significance. **(e)** Distribution of WGCNA module eigengene scores across annotated immune-cell populations. **(f)** Gene Ontology enrichment analysis of Brown module genes. **(g)** UMAP visualization of CD45^+^ immune cells from Sham, 24 h and 72 h post-SAH samples, showing temporal remodeling of the immune-cell landscape after SAH. **(h)** Venn diagram illustrating the overlap between the 24-h Turquoise module and the top 500 marker genes of the N_04 neutrophil subcluster.
